# American Society of Plastic Surgeons Member Post-Operative Opioid Prescribing Patterns

**DOI:** 10.1097/GOX.0000000000002125

**Published:** 2019-03-13

**Authors:** Radbeh Torabi, Lynn Bourn, Gerhard S. Mundinger, Fouad Saeg, Charles Patterson, Alejandro Gimenez, Ian Wisecarver, Hugo St. Hilaire, Mark Stalder, Oren Tessler

**Affiliations:** From the * Department of Surgery, Section of Plastic & Reconstructive Surgery, Louisiana State University Health Sciences Center, New Orleans, La.; † School of Medicine, Louisiana State University Health Sciences Center, New Orleans, La.; ‡ School of Medicine, Tulane University, New Orleans, La.; § Elite Plastic Surgery, Phoenix, Ariz.

## Abstract

**Introduction:**

Despite the widespread use of opioids in pain management, there are currently no evidence-based guidelines for the treatment of postoperative pain with opioids. Although other surgical specialties have begun researching their pain prescribing patterns, there has yet to be an investigation to unravel opioid prescribing patterns among plastic surgeons.

**Methods:**

Survey Monkey was used to sample the American Society of Plastic Surgeons (ASPS) members regarding their opioid prescribing practice patterns. The survey was sent randomly to 50% of ASPS members. Respondents were randomized to 1 of 3 different common elective procedures in plastic surgery: breast augmentation, breast reduction, and abdominoplasty.

**Results:**

Of the 5,770 overall active ASPS members, 298 responses (12% response rate) were received with the following procedure randomization results: 106 for breast augmentation, 99 for breast reduction, and 95 for abdominoplasty. Overall, 80% (N = 240) of respondents used nonnarcotic adjuncts to manage postoperative pain, with 75.4% (N = 181) using nonnarcotics adjuncts >75% of the time. The most commonly prescribed narcotics were Hydrocodone with Acetaminophen (Lortab, Norco) and Oxycodone with Acetaminophen (Percocet, Oxycocet) at 42.5% (N = 116) and 38.1% (N = 104), respectively. The most common dosage was 5 mg (80.4%; N = 176), with 48.9% (N = 107) mostly dispensing 20–30 tablets, and the majority did not give refills (94.5%; N = 207).

**Conclusions:**

Overall, plastic surgeons seem to be in compliance with proposed American College of Surgeon’s opioid prescription guidelines. However, there remains a lack of evidence regarding appropriate opioid prescribing patterns for plastic surgeons.

## INTRODUCTION

Despite the widespread use of opioids in pain management, there are currently no evidence-based guidelines for the treatment of postoperative pain with opioids. According to data collected by the American College of Surgeons (ACS) National Surgical Quality Improvement Program, 9 out of 10 surgical patients received an opioid prescription at discharge.^1^ Meanwhile, 80% of patients complain of undertreated postoperative pain, with 75% describing the pain as moderate, severe, or extreme.^2^ These data highlight the inadequacy of employing a “one-size-fits-all,” opioid-based protocol which often leads to significant variation in the type of narcotic, the number of pills dispensed, and the dosage prescribed.^3^ In 2014, Current Concepts in Pain Management in Plastic Surgery combined a multidisciplinary approach from multiple articles in regards to treating pain. The supplement focused on a multimodal analgesic approach with opioids as an adjunctive role as opposed to the primary treatment modality.^4^

Alarmingly, the number of opioid prescriptions has increased by 400% from 1999 to 2014.^5^ However, the amount of pain reported by American patients has remained constant.^6,7^ The opioid epidemic is more prevalent today than ever before, with over 5 million Americans abusing opioids, resulting in >33,000 deaths annually and an economic burden of $78.5 billion per year.^8–10^ Over 70% of opioid abusers receive drugs through inappropriately obtained prescriptions, with more than half obtaining drugs from a family member or friend with excess pills.^11,12^ Despite their popularity in the treatment of postsurgical pain, opioid prescriptions written by surgeons often go unused. Surgeons prescribe 2–5 times more opioids than consumed, and 83% of patients claim to finish only half of the prescription.^13,14^ This phenomenon directly contributes to our nation’s opioid epidemic and puts the patient, their family, and the community at risk.^15^

Although other surgical specialties have begun researching their pain prescribing patterns, there has yet to be an investigation to unravel the patterns among plastic surgeons. The purpose of this study is to determine pain management regimens used by board-certified plastic surgeons in the United States and how this correlates to their practice or demographic characteristics, including trends in narcotic prescriptions and pain management education.

## METHODS

Survey Monkey (SurveyMonkey Inc., San Mateo, Calif.; website: www.surveymonkey.com) was used to sample the American Society of Plastic Surgeons (ASPS) members regarding their opioid prescribing practice patterns. The survey was sent randomly to 50% of ASPS members. The survey contained 67 questions, and respondents were randomized to 1 of 3 different invasive procedures that are some of the most common in plastic surgery based on ASPS statistics: breast augmentation, breast reduction, and abdominoplasty.^16^ Page randomization and branching logic was used so that when each respondent started a survey, they were randomized to 1 of the 3 procedures, to answer specific procedure questions, then all were redirected to the same questions directed at their overall practice. Questions for each procedure ascertained the frequency of use of nonnarcotic adjuncts and the narcotic prescribing patterns. Nonnarcotic adjuncts being described as either liposomal bupivacaine (Exparel; Pacira, Parsippany, N.J.), other long-acting anesthetic agent (bupivacaine, levobupivacaine, ropivacaine), intravenous acetaminophen (Ofirmev; Mallinckrodt Pharmaceuticals, Staines-upon-Thames, United Kingdom), or Ketorolac (Toradol; Hoffmann-La Roche, Switzerland). Afterwards, each respondent subsequently answered questions in the following categories: prescribing narcotic patterns, changes in prescribing patterns in the last 5–10 years, practice characteristics, institutional characteristics, narcotic training/education, and narcotic patient education. Survey questions are presented in Table 1. Data were analyzed for comparison between procedures and correlation between physician or institutional characteristics and prescribing patterns by means of chi-square and Fisher exact tests (StataCorp, 2015, Stata Statistical Software: Release 14; StataCorp LP, College Station, Tex.). Multivariate analysis was not performed due to insufficient univariate significance of predictor variables.

**Table 1.**
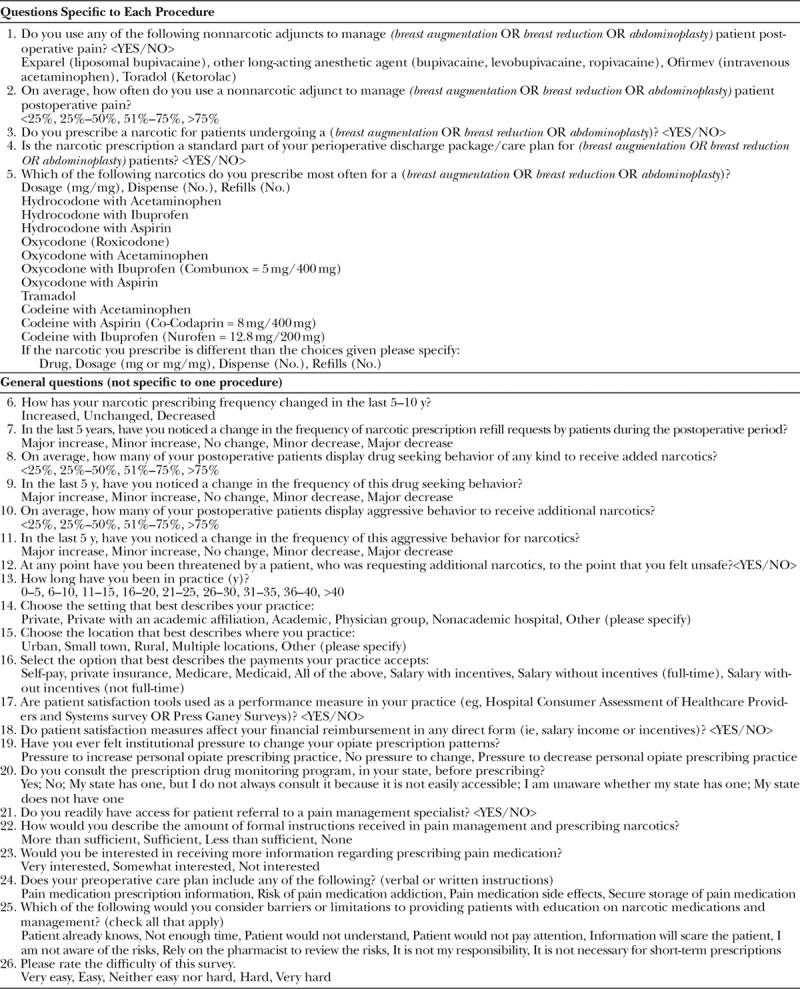
List of Survey Questions with Answer Choices

## RESULTS

### Demographics and Physician/Practice Characteristics

**Fig. 1.**
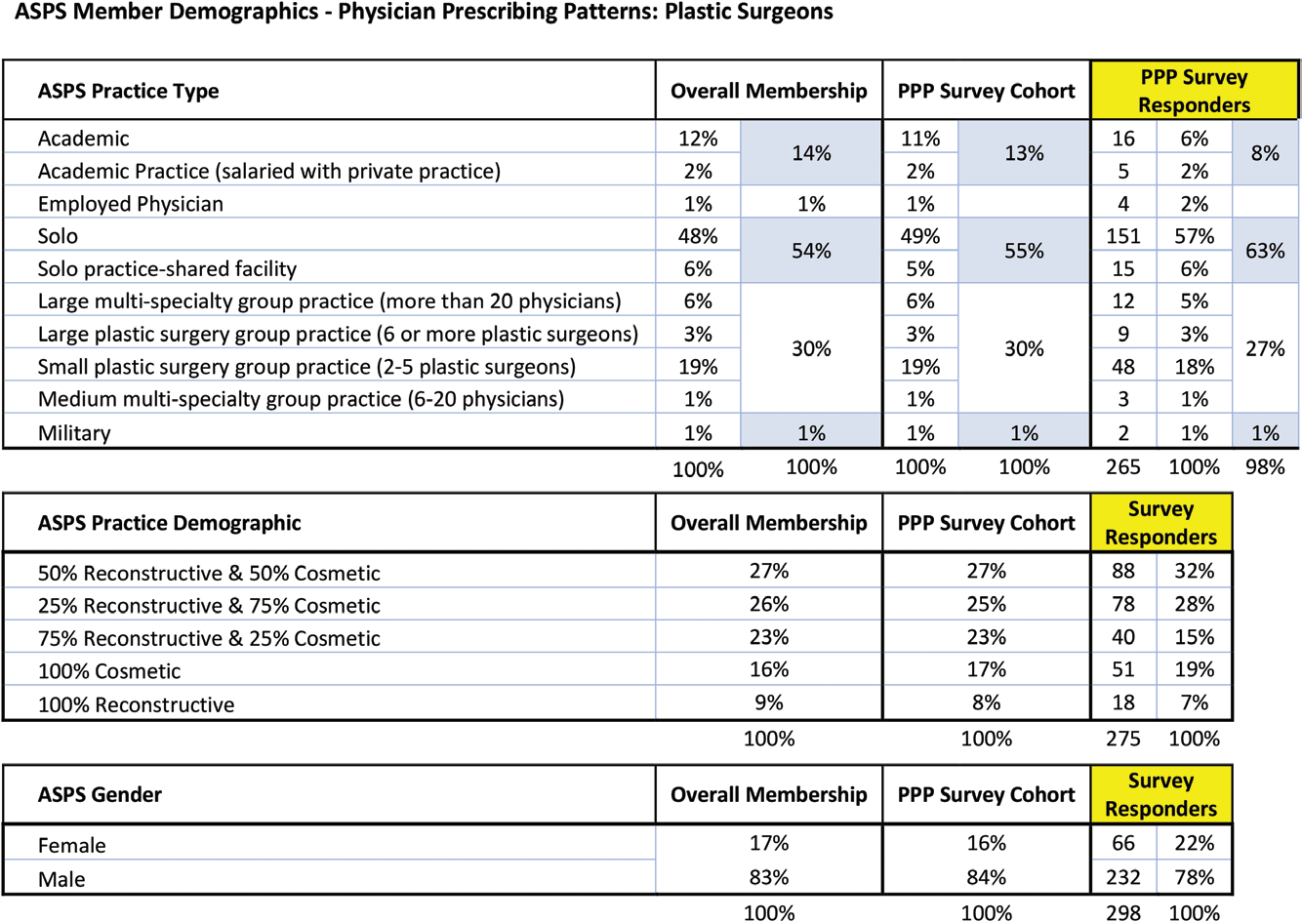
ASPS member demographics—physician prescribing patterns: plastic surgeons.

Of the randomly surveyed 2,885 ASPS members (50% of 5,770 total members), 298 responses were received (12% response rate). Upon comparing the respondent sample with self-reported demographic information for all ASPS members (Fig. 1), the only significant differences were that there was a larger number of respondents who were males (N = 232, 78%; *P* = 0.0067) who were in solo practice settings (N = 151, 57%; *P* = 0.0144) and who performed varying combinations of cosmetic and reconstructive procedures in their practice (74%, N = 206; *P* = 0.0195). The following are demographic results from the survey. In terms of practice setting and its effects on prescription patterns, the majority of plastic surgeons (66.1%; N = 187) had a private practice setting in an urban (61.8%; N = 175) location. The surgeons (48.1% only; N = 136) accepted self-pay, whereas 40.3% (N = 114) accepted most forms of payment (self-pay, private insurance, Medicare, and Medicaid). There was no statistical difference between practice settings (*P* = 0.467) or location (*P* = 0.836) upon comparison to narcotic prescriptions for postoperative pain management. Self-pay-only plastic surgeons had a 100% rate of narcotic use as part of their discharge packets. This was significantly higher than those who accept all forms of payment (*P* = 0.035). However, self-pay plastic surgeons were no different in the amount of nonnarcotic prescriptions used (*P* = 0.100) or narcotic prescriptions used (*P* = 0.161) to manage postoperative pain.

### Prescribing Patterns by Procedure: Breast Augmentation, Breast Reduction, Abdominoplasty

**Fig. 2.**
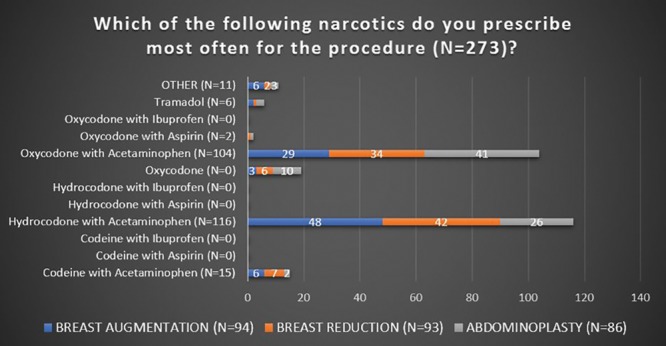
Response of plastic surgeons to what narcotic prescriptions were given for each procedure (breast augmentation, breast reduction, abdominoplasty).

**Fig. 3.**
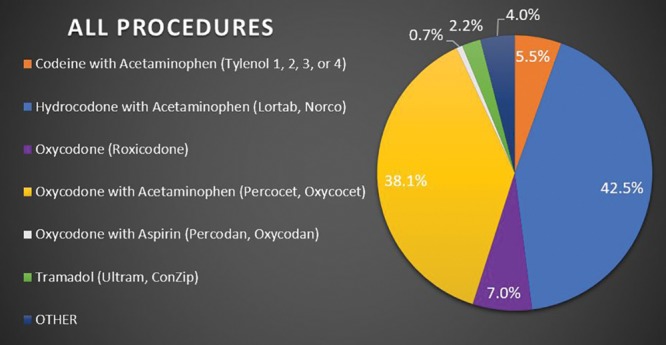
Response of plastic surgeons to what narcotic prescriptions were given most often for all procedures combined.

**Fig. 4.**
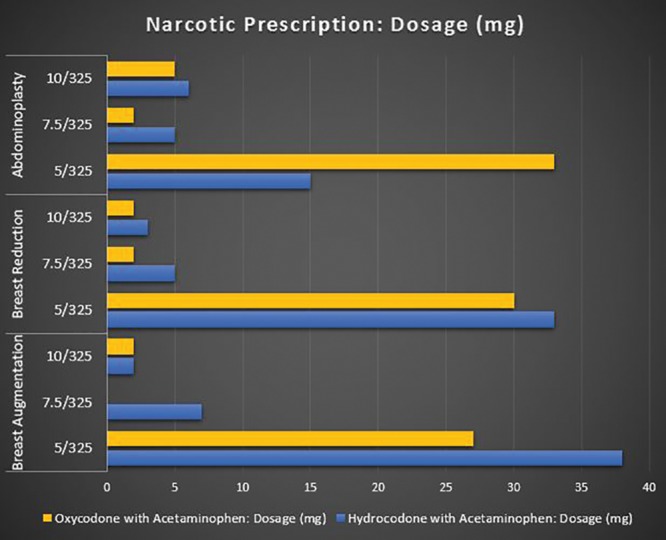
Narcotic prescription dosage (mg) of oxycodone and hydrocodone with acetaminophen by procedure.

**Fig. 5.**
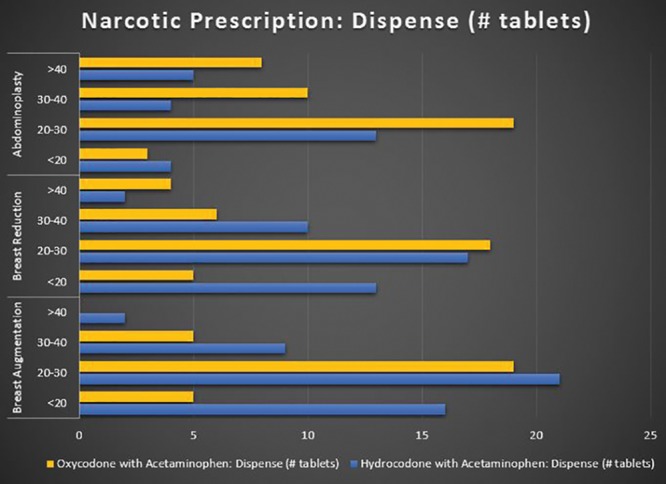
Narcotic prescription number of tablets dispensed of oxycodone and hydrocodone with acetaminophen by procedure.

Survey respondents were randomized as follows: 106 for breast augmentation, 99 for breast reduction, and 95 for abdominoplasty. Overall, 80% (N = 240) of respondents used nonnarcotic adjuncts to manage postoperative pain; with 75.4% (N = 181) using nonnarcotics adjuncts >75% of the time. The respondents (92.6%; N = 296) prescribed a narcotic for postoperative pain, with the prescription being a standard part (96.4%; N = 277) of their perioperative discharge package/care plan for the procedure. The most commonly prescribed narcotics were Hydrocodone with Acetaminophen (UCB Pharma, Brussels, Belgium) and Oxycodone with Acetaminophen (Endo Pharmaceuticals, Dublin, Ireland) at 42.5% (N = 116) and 38.1% (N = 104), respectively. Out of these 220 plastic surgeons, the most common dosage was 5 mg (80.4%; N = 176), with 48.9% (N = 107) mostly dispensing 20–30 tablets and the majority did not give refills (94.5%; N = 207). See Figures 2–5 for full graphical comparison. Upon statistical comparison between procedures, there was no significant variation in the use of nonnarcotic adjuncts (*P* = 0.646) or narcotic prescription use (*P* = 0.463).

### Changes to Prescribing Patterns over the Last 5–10 Years

Changes in prescribing patterns and postoperative patient characteristics were as follows: overall 56.9% (N = 152) felt their narcotic prescribing frequency had decreased in the last 5–10 years, whereas 41.6% (N = 111) described it as unchanged. Although the majority of plastic surgeons did not notice a change (63.7%; N = 170) in the frequency of narcotic prescription refill requests by patients, some felt there to be a minor increase (11.2%) or minor decrease (13.5%). Physicians felt that <25% of patients displayed drug seeking (97%; N = 259) or aggressive (99.3%; N = 265) behavior of any kind to receive added narcotics.

### Prescribing Patterns and Patient Satisfaction Tools

Patient satisfaction tools (eg, Hospital Consumer Assessment of Healthcare Providers and Systems survey or Press Ganey Surveys) were used as a performance measure in 50.9% (N = 144) of practices. For the majority of plastic surgeons, patient satisfaction measures did not affect financial reimbursement in any direct form (ie, salary income or incentives; 90.8%; N = 257). For those reporting use of patient satisfaction tools as a performance measure in their practice, there was no significant variation in use of nonnarcotic pain medication (*P* = 0.467) or narcotic prescriptions (*P* = 0.417). For those plastic surgeons in whom patient satisfaction measures affected their financial reimbursement, there was no significant variation in use of nonnarcotic pain medication (*P* = 0.267) or narcotic prescriptions (*P* = 0.573).

### Prescription Drug Monitoring Programs

The respondents (31.2%; N = 88) reported consulting their state’s prescription drug monitoring program (PDMP) before prescribing narcotics, whereas 36.2% (N = 102) do not. Plastics surgeons (22.3%; N = 63) did have a state PDMP but did not always consult it because they felt it was not easily accessible. Plastic surgeons (78.4%; N = 221) reported that they readily have access for patient referral to a pain management specialist. Only 17% (N = 48) of plastic surgeons described the amount of formal instructions they received in pain management and prescribing narcotics to be less than sufficient.

### Pain Medication Patient Education

In regard to their preoperative care plan (verbal or written instructions), plastic surgeons provided the following: pain medication prescription information (88%; N = 243), risk of pain medication addiction (45.3%; N = 124), pain medication side effects (80.4%; N = 221), and secure storage of pain medication (36.8%; N = 100). The most commonly reported barrier or limitation to providing patients with education on narcotic medications and management was that the “patient won’t pay attention” (52.9%; N = 146); which was followed by “not enough time” (36.2%; N = 100) and “unnecessary for short term prescriptions” (34.1%; N = 94).

### Prescribing Patterns and Years in Practice

Upon comparison of physician experience, there was no significant variation in use of nonnarcotic pain medication (*P* = 0.672) or narcotic prescriptions (*P* = 0.213) for those in practice <10 years versus those >10 years. In addition, most plastic surgeons felt no pressure to change personal opiate prescribing practice (76.3%; N = 216), although some felt pressure to decrease (21.9%; N = 62).

## DISCUSSION

### Opioid Use in Surgical Specialties

It is increasingly important for surgeons, emergency room and primary care providers, and their patients to understand the detrimental effects associated with opioid use. Nevertheless, prescription opioids remain an important analgesic in the treatment of acute postsurgical pain. The surgical specialties including general, orthopedic, plastic, cardiothoracic, vascular, colorectal, spinal, and neurologic prescribe opioid analgesics at a rate of 36.5% which accounts for 10% of the total opioid prescriptions in the United States.^17^ Despite their popularity in the treatment of postsurgical pain, opioid prescriptions written by surgeons often go unused. Surgeons prescribe 2–5 times more opioids than consumed, and 83% of patients claim to finish only half of the prescription.^13,14^ This may, however, be affected by patients reporting less than the actual amount consumed. In addition, 3 of 4 postoperative patients fail to lock stored narcotics or properly dispose of left over medications.^18^ Specifically, breast augmentation and breast reduction patients report an average of 12–18 unused pain pills postoperatively.^19^ However, the trend seen among surgical specialties from 2007 to 2012 indicates a decreasing pattern of opioid prescribing.^17^ This is perhaps due to an increase in surgeons adopting the use of multimodal analgesia to treat postsurgical pain. In plastic surgery, multimodal analgesics have been shown to reduce opioid use and effectively treat postsurgical pain.^20,21^ Regular use of acetaminophen, ibuprofen, and celecoxib in plastic surgery patients without contraindications has been shown to provide sufficient pain control and reduce opioid requirements postoperatively.^22–25^

### Suggested Opioid Prescribing Guidelines for Surgeons

The Center for Disease Control issued guidelines for prescribing narcotics for chronic pain, but none exist for acute postoperative pain; however, surgeons operate on patients with chronic pain syndromes.^26^ Chronic pain guidelines included the following: nonpharmacologic therapy and nonopioid pharmacologic combined with opioid pharmacologic therapy, establishing realistic goals for pain and function, discussing risks and realistic benefits of opioid therapy, avoiding extended-release/long-acting opioids, using the lowest effective dosage for 3–7 days, review of the patient’s history of controlled substance prescriptions using state PDMP, and avoiding opioid pain medication and benzodiazepines concurrently when possible.

Although no opioid guidelines exist for plastic surgeons, the ACS support state PDMP, prescribing limits, the use of nonopioid alternatives, continuing medical education on opioid-specific training.^27^ The American Pain Society, in collaboration with the American Society of Anesthesiologists, developed an evidence-based guideline on postoperative pain management. Key recommendations included multimodal technique (combining opioid and nonopioid medications) and local or regional anesthetic blocks.^2^ Many states have recently instituted changes to opioid prescribing laws but they vary widely. For example, mandates in Louisiana for 2018 included limits to duration of narcotic treatment with required continuing education in controlled substances and addiction treatment.^28^ Based on results of this ASPS survey, plastic surgeons seem to be appropriate in their prescribing patterns of narcotics with the majority giving lower dosages and enough pills to last no longer than a week with no refills. Their prescribing patterns were not affected by variation in procedure, practice setting or location, or number of years in practice. However, self-pay-only plastic surgeons all included a standard prescription as part of their perioperative discharge care package. Many plastic surgeons utilize nonnarcotic adjuncts, in addition to opioid, to manage postoperative pain which show an attempt for combination therapy pain management approach in line with ACS guidelines.

### Use of State PDMP

One third of plastic surgeons consult their state’s PDMP, whereas one third do not. Almost one fourth of plastics surgeons felt their state’s drug monitoring was not easily accessible and thus hindered the consult; however, most plastic surgeons readily have access to a pain management specialist. Although PDMP are available in almost every state, studies show they did not allow or foster access by health care professionals. The U.S. Department of Health and Human Services, in recent years, began funding the “Enhancing Access to Prescription Drug Monitoring Programs” project to aid in increasing physician access to services.^29^ Although the procedures in this survey are cosmetic, and not subject to rules regarding Medicare reimbursement, plastic surgeons were answering overarching practice characteristic questions. Consulting PDMP is listed as one of the most relevant improvement activities related to Medicare reimbursements.^30^ Therefore, ASPS education regarding Merit-Based Incentive Payment System could explain the increase in plastic surgeon reporting in comparison to the published surgeon national average consulting for PDMP.

### Pain Medication and Patient Education

Although one third of plastic surgeons felt pain medication education was unnecessary for short-term pain prescriptions, those that did provide education mainly focused on the prescription and side effects. Only half talked to the patient about addiction and a third mentioned safe storage. Although <20% of plastic surgeons felt amount of formal instructions received in pain management and prescribing narcotics to be inadequate, it is possible that plastic surgeons need more education in providing opioid patient education; however, the most common reported barrier to education for plastic surgeons was the patient will not pay attention. Attempting to educate the day of the surgery has been shown to be less effective than during a preoperative appointment. This could be the reason why plastic surgeons felt patients do not pay attention.^31^ Although the survey revealed most plastic surgeon care plans included pain medication prescription information and pain medication side effects, less than half provided risk of pain medication addiction and secure storage of pain medication. The ASPS highlighted an article regarding tips on how plastic surgeons can manage opioid addiction risk. Suggested strategies for plastic surgeons included screening for risk factors of opioid use disorder, talking to patients about these risks in a nonjudgmental way, and informing the patient’s primary care doctor about the possible increase in risk. In addition, referrals to an addiction specialist, before surgery, is recommended for patients with known or suspected substance use disorder. Neither the ASPS nor the ACS has specific guidelines regarding drug screening, but remaining alert to verbal and nonverbal clues will help guide physical examination to confirm suspicion.^31^ ASPS also promoted methods to reduce the need for narcotics such as anesthesia techniques and nonopioid pain management strategies.^32^

### Pain Management Recommendations by Procedure

In regards to plastic surgery patients, elective procedures were associated with higher rates of prolonged opioid use than trauma-related surgeries.^33^ Muscle relaxers are recommended for abdominoplasties in conjunction with patient education regarding positioning and techniques to decrease strain to abdominal wall.^34^ In addition, the use of epidural pain management reduced the need for opioids and shortened hospital stay for abdominoplasties.^35^ On the same note, the use of self-adhering mesh intraoperatively was associated with significantly less acute postoperative pain.^36^ The use of nonnarcotic analgesics, such as NSAIDS, for breast augmentation was associated with significantly lower pain during the first 3 postoperative days, earlier recovery of bowel function and normal activities, without increase in bleeding risk.^21^

### Limitations

Low response rate, 12% (298), is a limitation of this study. ASPS only allowed the survey to be sent random to 50% of their members. A PubMed search for “ASPS survey response” yielded 13 articles with an average reported survey response rate of 14.7%; thus, our ASPS member response rate of 12% is externally valid for the population sampled. Upon further investigation, one article looked at 1,607 different surveys from multiple academic journals and determined the average response rate of data collected from individuals to be 52.7% with an SD of 20.4.^37^ As a whole, the low response rates of ASPS member surveys subjects results to nonresponse bias. Performing a nonresponse analysis, to compare survey respondents with nonrespondents, allows for assessment of potential bias in results.^38^ Our study focused on board-certified plastic surgeons and did not look at physicians practicing outside the scope of their board practice. Future studies could survey the prescribing practices of a broad array of physicians, in relation to cosmetic procedures performed, through the aid of ACS or American Medical Association. Another limitation of this study could be insufficient power, despite 298 responses, to detect differences in narcotic and nonnarcotic patterns in relation to practice setting and physician characteristics. This affects the ability to generalize results to all plastic surgeons.

Two hundred and sixty-six of 298 respondents (89.3%) fully completed survey. Due to some respondents with partial survey completion, 32 of 298 respondents answered questions about narcotic and nonnarcotic prescribing patterns only. Therefore, this affected the number of responses regarding practice and physician characteristics. Due to the structure of the survey, it is not feasible to compare procedures to practice settings and physician characteristics. This is because after the randomization to a procedure, all respondents were directed to the same pages for the second half of the survey. The questions on this section were not specific to any procedure. In addition, the data collected through the survey were distributed to us for analysis directly from ASPS. This did not include practice characteristics separated by assigned procedure. This was not accounted for in data analysis. Geographical distribution of plastic surgeon respondents was not collected. This information could have added to the article, with the ability to report geographic tendencies with narcotic prescribing patterns.

## CONCLUSIONS

Overall, plastic surgeons seem to be in compliance with proposed ACS opioid prescription guidelines: support state PDMP, prescribing limits, the use of nonopioid alternatives, continuing medical education on opioid- specific training. However, there remains a lack of evidence regarding appropriate opioid prescribing patterns for plastic surgeons. This study brings awareness to the need for a large prospective study, which includes both plastic surgeon and patient responses, to develop plastic surgery prescribing guidelines.

## References

[1] Thiels CA Anderson SS Ubl DS Wide variation and overprescription of opioids after elective surgery. Ann Surg. 2017;266:564–573. 28697049 10.1097/SLA.0000000000002365

[2] Chou R Gordon DB de Leon-Casasola OA Management of postoperative pain: a clinical practice guideline from the American Pain Society, the American Society of Regional Anesthesia and Pain Medicine, and the American Society of Anesthesiologists’ Committee on Regional Anesthesia, Executive Committee, and Administrative Council. J Pain. 2016;17:131–157. 26827847 10.1016/j.jpain.2015.12.008

[3] Hill MV McMahon ML Stucke RS Wide variation and excessive dosage of opioid prescriptions for common general surgical procedures. Ann Surg. 2017;265:709–714. 27631771 10.1097/SLA.0000000000001993

[4] Janis JE Joshi GP Introduction to “current concepts in pain management in plastic surgery.” Plast Reconstr Surg. 2014;134(4 suppl 2):6S–7S. 10.1097/PRS.0000000000000683 25255010

[5] Centers for Disease Control and Prevention. Opioid overdose. Available at https://www.cdc.gov/drugoverdose/index.html. Accessed April 16, 2018.

[6] Daubresse M Chang HY Yu Y Ambulatory diagnosis and treatment of nonmalignant pain in the United States, 2000–2010. Med Care. 2013;51:870–878. 24025657 10.1097/MLR.0b013e3182a95d86 PMC3845222

[7] Chang H-Y Daubresse M Kruszewski SP Prevalence and treatment of pain in emergency departments in the United States, 2000–2010. Pharmacoepidemiol Drug Saf. 2014;23:114. 10.1016/j.ajem.2014.01.015 24560834

[8] Manchikanti L Helm S Fellows B Opioid epidemic in the United States. Pain Physician. 2012;15(3 suppl):ES9–E38. 22786464

[9] Rudd RA Seth P David F Increases in drug and opioid-involved overdose deaths—United States, 2010–2015. MMWR Morb Mortal Wkly Rep. 2016;65:1445–1452. 28033313 10.15585/mmwr.mm655051e1

[10] Florence CS Zhou C Luo F The economic burden of prescription opioid overdose, abuse, and dependence in the United States, 2013. Med Care. 2016;54:901–906. 27623005 10.1097/MLR.0000000000000625 PMC5975355

[11] Chen JH Humphreys K Shah NH Distribution of opioids by different types of medicare prescribers. JAMA Intern Med. 2016;176:259–261. 10.1001/jamainternmed.2015.6662 PMC5374118 26658497

[12] Maxwell JC The prescription drug epidemic in the United States: a perfect storm. Drug Alcohol Rev. 2011;30:264–270. 21545556 10.1111/j.1465-3362.2011.00291.x

[13] Bartels K Mayes LM Dingmann C Opioid use and storage patterns by patients after hospital discharge following surgery. PLoS One. 2016;11:e0147972. 26824844 10.1371/journal.pone.0147972 PMC4732746

[14] Gauger EM Gauger EJ Desai MJ Opioid use after upper extremity surgery. J Hand Surg Am. 2018;43:470–479. 29602656 10.1016/j.jhsa.2018.02.026

[15] Hooten WM St Sauver JL McGree ME Incidence and risk factors for progression from short-term to episodic or long-term opioid prescribing: a {population-based} study. Mayo Clin Proc. 2015;90:850–856. 26141327 10.1016/j.mayocp.2015.04.012 PMC4548808

[16] American Society of Plastic Surgeons. 2017 Plastic Surgery Statistics. Available at https://www.plasticsurgery.org/news/plastic-surgery-statistics. Accessed May 2, 2018.

[17] Levy B Paulozzi L Mack KA Trends in opioid analgesic-prescribing rates by specialty, U.S., 2007–2012. Am J Prev Med. 2015;49:409–413. 25896191 10.1016/j.amepre.2015.02.020 PMC6034509

[18] Bicket MC Long JJ Pronovost PJ Prescription opioid analgesics commonly unused after surgery: a systematic review. JAMA Surg. 2017;152:1066–1071. 28768328 10.1001/jamasurg.2017.0831 PMC5701659

[19] Hart AM Broecker J Kao L Opioid usage following outpatient breast surgery: are physicians part of the problem? Plast Reconstr Surg. 2018;142:611–620. 29878998 10.1097/PRS.0000000000004636

[20] Lalonde D Wong A Local anesthetics: what’s new in minimal pain injection and best evidence in pain control. Plast Reconstr Surg. 2014;134(4 suppl 2):40S–49S. 25255006 10.1097/PRS.0000000000000679

[21] Low YH Gan TJ NMDA receptor antagonists, gabapentinoids, α-2 agonists, and dexamethasone and other non-opioid adjuvants: do they have a role in plastic surgery? Plast Reconstr Surg. 2014;134(4 suppl 2):69S–82S. 25255009 10.1097/PRS.0000000000000703

[22] Simmons DL Wagner D Westover K Nonsteroidal anti-inflammatory drugs, acetaminophen, cyclooxygenase 2, and fever. Clin Infect Dis. 2000;31(suppl 5):S211–S218. 11113025 10.1086/317517

[23] Mitchell A McCrea P Inglis K A randomized, controlled trial comparing acetaminophen plus ibuprofen versus acetaminophen plus codeine plus caffeine (Tylenol 3) after outpatient breast surgery. Ann Surg Oncol. 2012;19:3792–3800. 22713999 10.1245/s10434-012-2447-7

[24] Manassa EH Hellmich S Ronert M Pain management after lipoplasty: a study of 303 cases. Plast Reconstr Surg. 2005;115:1715–1721; discussion 1722. 15861079 10.1097/01.prs.0000161453.43037.fa

[25] Sun T Sacan O White PF Perioperative versus postoperative celecoxib on patient outcomes after major plastic surgery procedures. Anesth Analg. 2008;106:950–958, table of contents. 18292445 10.1213/ane.0b013e3181618831

[26] Marcusa DP Mann RA Cron DC Prescription opioid use among opioid-naive women undergoing immediate breast reconstruction. Plast Reconstr Surg. 2017;140:1081–1090. 29176408 10.1097/PRS.0000000000003832

[27] Saluja S Selzer D Meara JG The opioid epidemic: what can surgeons do about it? Available at http://bulletin.facs.org/2017/07/the-opioid-epidemic-what-can-surgeons-do-about-it/#.WuJ9GIjwbIU. Published July 1, 2017. Accessed April 26, 2018. 28884995

[28] Louisiana State Board of Medical Examiners. Opioid Prescribing Laws—Recent Changes. Available at http://www.lsbme.la.gov/content/opioid-prescribing-laws-recent-changes. Published January 4, 2018. Accessed January 13, 2018.

[29] Wilsey B Prasad H Real-time access to prescription drug monitoring databases. CMAJ. 2012;184:1767–1768. 23008488 10.1503/cmaj.121495 PMC3494350

[30] American Society of Plastic Surgeons. Participate in MIPS: Improvement Activities Performance Category. Available at https://www.plasticsurgery.org/for-medical-professionals/health-policy/macra/participate-in-mips/mips-ia. Accessed June 10, 2018.

[31] Cone JD Jr Harrington MA Kelley SS Drug abuse in plastic surgery patients: optimizing detection and minimizing complications. Plast Reconstr Surg. 2011;127:445–455. 20871481 10.1097/PRS.0b013e3181fad5ac

[32] Demsey D Carr NJ Clarke H Managing opioid addiction risk in plastic surgery during the perioperative period. Plast Reconstr Surg. 2017;140:613e–619e. 10.1097/PRS.0000000000003742 PMC5783634 28953743

[33] Johnson SP Chung KC Zhong L Risk of prolonged opioid use among opioid-naïve patients following common hand surgery procedures. J Hand Surg Am. 2016;41:947–957.e3. 27692801 10.1016/j.jhsa.2016.07.113

[34] Constantine FC Matarasso A Putting it all together: recommendations for improving pain management in body contouring. Plast Reconstr Surg. 2014;134(4 suppl 2):113S–119S. 25254993 10.1097/PRS.0000000000000669

[35] Khansa I Koogler A Richards J Pain management in abdominal wall reconstruction. Plast Reconstr Surg Glob Open. 2017;5:e1400. 28740797 10.1097/GOX.0000000000001400 PMC5505858

[36] Khansa I Janis JE Abdominal wall reconstruction using retrorectus self-adhering mesh: a novel approach. Plast Reconstr Surg Glob Open. 2016;4:e1145. 27975037 10.1097/GOX.0000000000001145 PMC5142503

[37] Baruch Y Holtom BC Survey response rate levels and trends in organizational research. Human Relations. 2008;61:1139–1160.

[38] Johnson TP Wislar JS Response rates and nonresponse errors in surveys. JAMA. 2012;307:1805–1806. 22550194 10.1001/jama.2012.3532

